# Valproate-Induced Metabolic Syndrome

**DOI:** 10.3390/biomedicines11051499

**Published:** 2023-05-22

**Authors:** Natalia A. Shnayder, Violetta V. Grechkina, Vera V. Trefilova, Ilya S. Efremov, Evgenia A. Dontceva, Ekaterina A. Narodova, Marina M. Petrova, Irina A. Soloveva, Liia E. Tepnadze, Polina A. Reznichenko, Mustafa Al-Zamil, Gulnara I. Altynbekova, Anna I. Strelnik, Regina F. Nasyrova

**Affiliations:** 1 Institute of Personalized Psychiatry and Neurology, Shared Core Facilities, V.M. Bekhterev National Medical Research Centre for Psychiatry and Neurology, 192019 Saint Petersburg, Russia; grechkina.vv@mail.ru (V.V.G.); vera.v.trefilova@yandex.ru (V.V.T.); 2 Shared Core Facilities “Molecular and Cell Technologies”, V.F. Voino-Yasenetsky Krasnoyarsk State Medical University, 660022 Krasnoyarsk, Russia; shapo_jain@mail.ru (E.A.D.); katya_n2001@mail.ru (E.A.N.); stk99@yandex.ru (M.M.P.); solovieva.irina@inbox.ru (I.A.S.); liatepnadze@yandex.ru (L.E.T.); polina.reznichenko.98@mail.ru (P.A.R.); 3 Department of Neurology, Hospital for War Veterans, 193079 Saint Petersburg, Russia; 4 Department of Psychiatry and Narcology, Bashkir State Medical University, 450008 Ufa, Russia; efremovilya102@gmail.com; 5 Federal Centre for Neurosurgery, 630087 Novosibirsk, Russia; 6 Department of Physiotherapy, Faculty of Continuing Medical Education, Peoples’ Friendship University of Russia, 117198 Moscow, Russia; alzamil@mail.ru; 7 Department of Psychiatry and Narcology, S.D. Asfendiarov Kazakh National Medical University, Almaty 050022, Kazakhstan; profi.21@mail.ru; 8 International Centre for Education and Research in Neuropsychiatry, Samara State Medical University, 443016 Samara, Russia; a.i.strelnik@samsmu.ru

**Keywords:** valproic acid, metabolic syndrome, personalized approach, metabolomic, metabolome

## Abstract

Valproic acid (VPA) and its salts (sodium calcium magnesium and orotic) are psychotropic drugs that are widely used in neurology and psychiatry. The long-term use of VPA increases the risk of developing adverse drug reactions (ADRs), among which metabolic syndrome (MetS) plays a special role. MetS belongs to a cluster of metabolic conditions such as abdominal obesity, high blood pressure, high blood glucose, high serum triglycerides, and low serum high-density lipoprotein. Valproate-induced MetS (VPA-MetS) is a common ADR that needs an updated multidisciplinary approach to its prevention and diagnosis. In this review, we consider the results of studies of blood (serum and plasma) and the urinary biomarkers of VPA-MetS. These metabolic biomarkers may provide the key to the development of a new multidisciplinary personalized strategy for the prevention and diagnosis of VPA-MetS in patients with neurological diseases, psychiatric disorders, and addiction diseases.

## 1. Introduction

**Figure 1:**
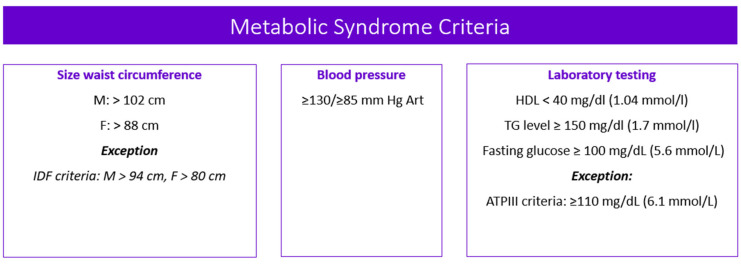
Metabolic syndrome criteria. Note: M—male; F—female; IDF—the International Diabetes Federation; ATPIII—the Adult Treatment Panel, III edition; HDL—high-density lipoprotein cholesterol; TG—triglycerides.

Metabolic syndrome (MetS) is the medical term for a combination of diabetes mellitus, arterial hypertension, and obesity [1,2,3,4]. It puts you at greater risk of getting cardiovascular and cerebrovascular diseases. The MetS criteria [5,6,7] are presented in Figure 1.

According to the Adult Treatment Panel, III edition (ATPIII) [8], the prevalence of this disorder is variable in different regions of the world, including: North America (USA)—23.7%; Europe—18.4% among male and 14.4% among female; and Asia—28.8% among male and 31.8% among female. In Russia, the prevalence of MetS is 20% or more in people of reproductive age, and in people over 60 years old it is about 47%, while in women it occurs 2.4 times more often [9,10,11].

**Figure 2:**
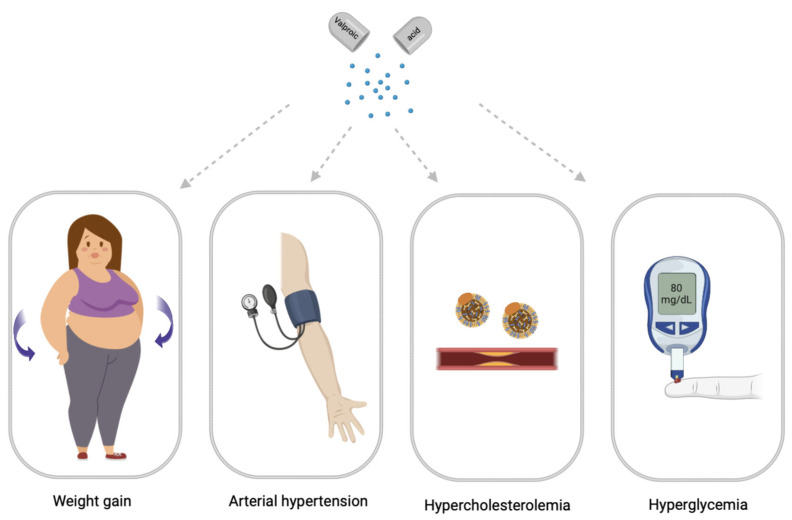
Main syndromes of valproate-induced metabolic syndrome (created with BioRender.com: https://www.biorender.com/ (accessed on 12 April 2023).

Drug-induced MetS is a combination of such conditions as abdominal obesity, hypercholesterolemia, hyperglycemia, and high blood pressure which occur while taking drugs [12]. Drug-induced MetS can be associated with various drugs, including psychotropic drugs [13]. Among psychotropic drugs, antipsychotics and valproic acid (VPA) drugs are the most common cause of drug-induced MetS [1,14] (Figure 2).

As is known, VPA (CH_3_CH_2_CH_2_)_2_CHCOOH 2-propylvaleric acid) is a psychotropic drug that is used in the treatment of many neurological diseases and mental disorders. The widespread use of VPA in neurology and psychiatry has led to an increased awareness of a spectrum of problematic and common adverse drug reactions (ADRs), including hepatotoxicity, nausea, vomiting, ataxia, lethargy, alopecia, thrombocytopenia, hyperammonemia, increased appetite, and weight gain. Among these ADRs, weight gain is of particular concern because the long-term use of valproates can lead to a significant increase in body weight, which is recorded in recent studies from 10% to 70% of patients and has been shown to lead to a number of metabolic disorders that can promote the development of VPA-induced MetS [15]. As a rule, the use of VPA is chronic, which increases the risk of developing VPA-induced MetS (VPA-MetS) [1,16]. The risk of developing VPA-MetS also depends on the genetic predisposition in a particular patient. This ADR may directly impair the quality of life of patients receiving valproate and be associated with a high risk of cardiovascular disease. From this position, both the prediction and prevention of the development of VPA-MetS and its early diagnosis are an urgent problem [14]. Significant weight gain is one of the most common problems with VPA in both children and adults [17]. In addition, the chronic use of valproates is associated with an increase in the level of fast plasma insulin (FPI), the development of insulin resistance, especially in a subgroup of children and women [18]. In addition to weight gain and insulin resistance, VPA-MetS is accompanied by dyslipidemia, arterial hypertension, and type 2 diabetes mellitus [15]. In recent years, there has been active research on: the pathophysiological mechanisms by which VPA and its active metabolites cause weight gain and metabolic disturbances, including increased thirst and hunger, caused by hypothalamic dysregulation due to an increased transmission of gamma-aminobutyric acid (GABA); the mechanisms of the VPA-induced modification of adipokines genes that encode the neuropeptides involved in central energy metabolism; and the VPA-induced deficiency of beta-oxidation of fatty acids [15].

Weight gain and metabolic disturbances associated with VPA intake have been investigated by various scientific groups and are of great concern to clinicians. The development of VPA-MetS reduces the quality of life of patients with neurological diseases and mental disorders, suffocates adherence to the exposure regimen, and leads to an increase in the incidence of cardiovascular and endocrine diseases. However, the diagnosis of VPA-MetS in the early stages of its development can be difficult due to the lack of unified approaches to the definition of this ADR and the methodology for using the use of VPA-MetS biomarkers in the biological fluids of the human body, primarily in the blood and urine. These reasons explain the relevance of this descriptive review, which summarizes the results of new studies of blood (serum and plasma) and urinary VPA-MetS biomarkers from the perspective of pharmacometabolomics.

## 2. Main Clinical Symptoms of Valproate-Induced Metabolic Syndrome

### 2.1. Valproate-Induced Weight Gain

Weight gain, also known as central obesity, is characterized by the excessive accumulation of fat in the abdomen and is associated with an increased risk of metabolic and cardiovascular diseases [19].

The features of abdominal obesity associated with VPA intake may include:

− An increase in body weight, body mass index (BMI), and waist circumference;
− An increase in the amount of fat in the abdomen, measured by the waist circumference or the ratio of waist to hips;
− Insulin resistance, which can contribute to the development of abdominal obesity.

Previous clinical studies have shown that weight gain is more common in women with epilepsy than in men. Therefore, gender can be considered one of the risk factors for VPA-induced weight gain [20]. In addition, the body fat percentage and waist-to-hip ratio are statistically significantly different between the sexes, with women having a higher body fat percentage and a lower waist-to-hip ratio. In female patients, an additional risk factor for VPA-induced weight gain is young age (weight gain is more common in post-pubertal girls taking VPA, and weight gain is more common in patients receiving VPA during puberty if epilepsy and therapy continue into adulthood) [20]. In adolescent girls, VPA-induced weight gain not only has serious psychological consequences, but can also lead to the development of important endocrinological abnormalities and reduced compliance with antiepileptic therapy. The mechanism by which VPA may cause weight gain is a matter of debate. However, various hypotheses have been put forward to explain the effect of VPA on weight gain: dysregulation of the hypothalamic system; effects on adipokines; hyperinsulinemia; and insulin resistance [21].

Experimental data have shown that VPA can cause dysregulation of the hypothalamic system [22]. This theory can be explained by an increase in gamma-aminobutyric acid (GABA) transmission in the hypothalamic axis. This is supported by studies whereby patients with epilepsy were treated with VPA, reporting weight gain, experienced an increase in appetite, and satiation with high-calorie drinks [20].

Another hypothesis is that VPA may cause weight gain by altering the expression of the adipokines genes that are expressed in the brain (pituitary gland). These genes encode the neuropeptides involved in central energy metabolism, such as resistin and starvation-induced adipose tissue factor (also known as angiopoietin-like protein 4), which have become the main targets of VPA associated with the etiology of obesity, leptin synthesis, and the development of insulin resistance [23]. Although VPA can alter gene expression in the hypothalamus in vitro [24], it is not clear whether VPA has such effects in vivo [20]. VPA may affect adipokines released by adipose tissue such as adiponectin, leptin, the soluble leptin receptor, and ghrelin [20]. VPA can increase the expression of messenger ribonucleic acid (mRNA) adiponectin-binding receptors (adipoR1) in the human hepatoma cell line HepG2 [20,25]. Since adiponectin mRNA expression is known to decrease after VPA treatment in vivo [26], the increased expression of adipoR1 mRNA in liver cells may represent a favorable response to counterbalance the suppressed secretion of adiponectin from adipocytes. Alterations in this receptor/ligand expression balance may contribute to changes in fatty acid oxidation and the development of insulin resistance in VPA-induced weight gain.

### 2.2. Valproate-Induced Insulin Resistance

Insulin resistance is a pathological condition in which cells do not normally respond to insulin [27]. Insulin is a hormone that facilitates the transport of glucose from the blood into the cells, thus lowering blood glucose levels. Insulin is secreted by the pancreas in response to carbohydrates consumed in the diet. In insulin resistance, high insulin levels do not have the expected effect on glucose transport and blood sugar levels [28]. Insulin resistance can be caused by a variety of factors, including obesity, a sedentary lifestyle, high-fat diet, and certain diseases such as polycystic ovary syndrome, as well as ADRs with long-term VPA [16,29].

The mechanisms by which VPA causes insulin resistance are not yet fully understood, but are thought to be mediated by several factors. VPA has been shown to increase oxidative stress, inflammation, and endoplasmic reticulum stress, all of which may contribute to the development of insulin resistance [30]. In addition, it was found that VPA increases the expression of the *CEBP Alpha* and *SOCS-3*, *CEBP Alphain 3T3-L1* genes involved in the development of insulin resistance [31]. It was also found that VPA suppresses gluconeogenesis and glucagon expression, and also causes histopathological changes in the pancreas and liver [32]. The results of other studies give conflicting conclusions, including the hypothesis of an antidiabetic role of VPA in type II diabetes mellitus by modulating insulin signaling and gluconeogenesis mediated by thymus O1 protein [32]. Because VPA is a well-established drug, the detailed molecular mechanisms of the present findings may be explored for a possible new clinical application of valproate [32]. It is important to note that the relationship between VPA and insulin resistance is not yet conclusive, since there are studies in which the relationship between valproate intake and the development of insulin resistance has not been proven [33]. Further research is needed on this issue.

### 2.3. Valproate-Induced Arterial Hypertension

Arterial hypertension is a syndrome of increased systolic blood pressure from 140 mm Hg. Art. and above, and simultaneously or independently, diastolic blood pressure ≥90 mm Hg. Art. [34]. Arterial hypertension is one of the leading causes of death and affects the financial burden of the health care system in the world [35]. There are many studies that give conflicting results regarding the effect of VPA on the development of arterial hypertension [20].

In animal models, VPA has been shown to lower blood pressure in spontaneously hypertensive rats and prevent high-fat diet-induced hypertension [36]. VPA is known to have a broad mechanism of action, including blocking voltage-gated sodium channels, increasing GABA, and inhibiting the glutamate/N-methyl-D-aspartic acid (NMDA) receptors that mediate neuronal excitation. VPA can also influence the extracellular signal-related kinase (ERK) pathway [14]. Angiotensin leads to an increase in blood pressure by activating the ERK pathway in vascular smooth muscle cells [36,37]. On the other hand, it has been shown that under the action of angiotensin, phosphorylation and the activation of ERK occur. Since VPA can cause a decrease in ERK phosphorylation, this may be followed by a decrease in angiotensin-induced cardiac fibrosis. Therefore, it is possible that VPA also lowers blood pressure by reducing ERK phosphorylation. However, further studies of this mechanism of VPA influence on the reduction of ERK phosphorylation and blood pressure reduction are required [36,38].

Additionally, VPA acts as a direct inhibitor of histone deacetylase. Hyperacetylation of lysine residues on histones promotes the relaxation of deoxyribonucleic acid (DNA) and allows for an increase in gene transcription [1]. The long-term inhibition of histone deacetylase with VPA, regardless of the response to blood pressure, reduces hypertrophic, pro-inflammatory, and hypertensive responses by reducing reactive oxygen species and angiotensin II type 1 receptor expression in the heart, demonstrating the importance of uncontrolled histone deacetylase activity in arterial hypertension [14,39]. There are also reported cases of increased blood pressure after the initiation of valproate therapy [40].

### 2.4. Valproate-Induced Hypercholesterolemia

Hypercholesterolemia is a risk factor for the development of atherosclerosis [41]. Total cholesterol increases the risk of developing coronary heart disease by 2.52–3.20 times in reproductive age [42]. Several studies have also shown an increased mortality from atherosclerosis in people with epilepsy [43,44]. The effect of VPA on serum total cholesterol is still a matter of controversy. There is a significant change in lipid, lipoprotein and apolipoprotein profiles. A number of studies have demonstrated that in patients taking VPA long-term, there are no changes in the lipid profile induced by this anticonvulsant [43].

Finally, some studies show the effect of lowering serum total cholesterol levels with long-term valproate therapy [43,45]. The decrease in total cholesterol levels with the long-term use of VPA may be due to an increase in the activity of certain organs/organelles that also contribute to cholesterol metabolism. Since VPA can inhibit oxidative stress in the endoplasmic reticulum via the glycogen synthase kinase 3/β pathway, this may contribute to cholesterol metabolism and changes in the pathogenesis of atherosclerosis [43].

On the other hand, studies show that VPA can increase total and LDL cholesterol levels after 12 months of use [46]. The mean carotid intima-media thickness in patients with epilepsy treated with VPA was higher than in healthy controls. This proves that VPA can contribute to the development of atherosclerosis [47].

The mechanism of lipid profile changes under the influence of VPA and the risk of developing VPA-induced hypercholesterolemia are still unknown. A possible mechanism may be related to VPA-induced insulin resistance and hyperinsulinemia, which leads to impaired lipid transport and lipogenesis [48].

### 2.5. Valproate-Induced Hyperglycemia

Drug-induced hyperglycemia is a global problem, as it increases the risk of microvascular and macrovascular complications, infections, metabolic coma, and even death [49]. VPA inhibits class I (HDAC1, HDAC2, HDAC3, and HDAC8) and class II a (HDAC4, HDAC5, and HDAC7) histone deacetylase, resulting in the increased acetylation of H2, H3, and H4 histones, which alter the expression of their associated genes [14].

Recently, the use of VPA in various diseases has been investigated as a strategy for the reuse of clinically approved drugs [14]. On the other hand, there are reports that VPA reduces blood glucose and fat deposition in the adipose tissue and liver in mice and rats, and histone deacetylases classes I and IIa appear to be involved in the control of gluconeogenesis signaling and insulin production. On the other hand, VPA may reduce the microvascular complications of diabetes mellitus [50], requiring the mechanism of this drug response to be studied.

## 3. Blood Biomarkers (Serum and Plasma) of Valproate-Induced Metabolic Syndrome

A biomarker of MetS is a characteristic that is objectively measured and evaluated to identify this disorder in patients. The biomarkers of VPA-induced MetS are considered as prognostic tools or predictors for classifying and assessing disorder progression. They are used to monitor the clinical response to drugs, including VPA [1,14]. Traditionally, a laboratory diagnosis of VPA-MetS and assessment of the cardiovascular risk involves the analysis of blood (serum or plasma) biomarkers, including total cholesterol, TG, HDL-C, LDL-C, insulin, and C-peptide [51]. Metabolic overload causes oxidative stress. This leads to an imbalance between the formation and inactivation of reactive oxygen species (ROS). The ROS play an important role in many physiological systems. However, ROS contribute to cellular dysfunction under conditions of increased oxidative stress [52]. Oxidative stress may play an important role in the onset of symptoms associated with VPA-MetS (atherosclerosis, arterial hypertension, and insulin resistance) [52].

Let us consider the most studied and promising blood biomarkers of VPA-induced MetS.

### 3.1. Carbohydrates

#### Glucose

Glucose, also called dextrose, is one of a group of carbohydrates known as simple sugars (monosaccharides). Glucose is the main type of sugar in the blood and the main source of energy for the body’s cells. For most healthy people, normal blood sugar levels are as follows: from 4.0 to 5.4 mmol/L (72–99 mg/dL) on an empty stomach; up to 7.8 mol/L (140 mg/dL) 2 h after meals. One of the criteria of MetS is an increased fasting glucose level (>100 mg/dL) [5,53]. An increase in the level of glucose in the blood plasma is diagnosed in most patients with MetS [5].

The main reason for the increase in plasma glucose levels in patients taking valproates is insulin resistance. Although, some patients with insulin resistance who develop compensatory hyperinsulinemia may have normal glucose levels. When the function of β-cells of the islets of Langerhans of the pancreas decreases, compensatory mechanisms do not work. On the other hand, it has been demonstrated that hyperglycemia is not the first sign of VPA-Met-C, but develops as a late complication of chronic valproate therapy. Nevertheless, an early diagnosis of hyperglycemia in patients taking valproates is important because it increases the risk of secondary microvascular and macrovascular (cardiovascular and/or cerebrovascular) complications as a consequence of VPA-induced MetS. For example, one of the microvascular complications is diabetic neuropathy [54]. Microvascular disease may further accelerate the development of congestive heart failure and promote atherogenesis [55,56,57].

### 3.2. Acids

#### Uric Acid

Uric acid is the end product of cleavage of purine catabolism in the human body. Purines are nitrogenous bases in the DNA that form part of the structural framework of cells. Purine breakdown occurs when cells age and die, forming uric acid. Uric acid is also formed as a result of the metabolic breakdown of certain types of food, such as red meat, seafood, and legumes. The liver and intestinal mucosa produce most of the uric acid. The kidneys excrete two-thirds of uric acid, and the other third is excreted by the gastrointestinal tract [58]. The actual reference range of serum uric acid is estimated according to its variations in healthy people. Using this approach, serum uric acid values from 3.5 to 7.2 mg/dL in adult men and postmenopausal women and from 2.6 to 6.0 mg/dL in premenopausal women were recognized as normal in many countries. However, the normal values of serum uric acid may vary from laboratory to laboratory.

The causes of high uric acid levels (hyperuricemia) may be primary (elevated uric acid levels due to purine) or secondary (high uric acid levels due to another disease or condition, such as MetS). An increase in the level of this metabolic biomarker is observed in obesity. Moreover, the association with cardiovascular diseases and kidney diseases is obvious [59,60]. In recent years, there has been increasing evidence that uric acid can play an important pathophysiological role in many “cardio-nephrometabolic” disorders, which apparently do not depend on the deposition of sodium urate crystals, since this is also evident for serum uric acid concentrations below the saturation point with sodium urate. Uric acid exacerbates insulin resistance, hyperlipidemia, and fatty liver [61]. Uric acid also has a pronounced antioxidant effect [58]. Some studies suggest that elevated uric acid and homocysteine, together with oxidative stress, may contribute to atherosclerotic risk in patients taking long-term VPA [62]. At the same time, other studies show a decrease in plasma uric acid [63] in patients with VPA-MetS.

These results indicate the need to revise the concept of “asymptomatic” in chronic hyperuricemia and, consequently, to revise the normal range of serum uric acid levels. In light of the new scientific knowledge about the pathophysiological role of uric acid in human diseases in general and in VPA-MetS in particular, the threshold value of serum uric acid levels (<6.0 mg/dL or <360 mmol/L) seems to allow for better identification of the patients with VPA-induced MetS and should be reasonably taken into account for all patients taking valproates for a long time, especially in high daily doses.

### 3.3. Hormones

#### 3.3.1. Insulin

Insulin is an anabolic hormone. It promotes glucose uptake, glycogenesis, lipogenesis, and protein synthesis in the skeletal muscles and adipose tissue through the tyrosine kinase receptor pathway. In addition, insulin belongs to one of the most important factors in the regulation of glucose homeostasis in plasma, since it counteracts glucagon and other catabolic hormones—adrenaline, glucocorticoids, and growth hormone [64,65].

Hyperinsulinemia is a condition in which excessive insulin levels circulate in the blood compared to glucose levels. In the early stages of the development of insulin resistance, the pancreas can compensate for this by increasing insulin secretion into the bloodstream in an attempt to overcome defects in the peripheral action of insulin. At the same time, β-cells hypertrophy in response to the increased need of the human body for insulin. In conditions of starvation, basic compensation may be sufficient to maintain normal plasma glucose levels. However, after a meal, when glucose is rapidly absorbed from the intestine, a relative lack of insulin is detected due to inadequate compensation, since the fluctuations in glucose levels increase over time. Resistance to the biological effects of insulin is an important factor contributing to the development of MetS. The level of serum insulin may change against the background of chronic psychopharmacotherapy [5].

So, hyperinsulinemia can be the result of various metabolic diseases and conditions, including VPA-induced MetS. Hyperinsulinemia can be diagnosed by checking normal glucose levels that exceed 1.7 mmol/L (30 mg/dL) with an intramuscular administration of 1 mg of glucagon. Normal fasting insulin levels in healthy people are usually in the range of 3–25 mmol/L, which corresponds to approximately 0.05–0.42 mmol/L. However, in patients taking VPA, the insulin concentration can be significantly higher [66,67,68,69].

#### 3.3.2. Adiponectin

Adiponectin is a hormone synthesized and secreted by white adipose tissue, predominantly visceral adipocytes. Adiponectin is found in sufficient amounts in the blood (about 0.01% of the total plasma protein with a total concentration of about 5–10 µg/mL) [70]. Adiponectin is involved in the regulation of glucose levels and the breakdown of fatty acids. In humans, this protein is encoded by the *ADIPOQ* gene [71].

Adiponectin circulates in plasma in the form of a low molecular weight trimer, a medium molecular weight hexamer, and a high molecular weight 12–18-mer. However, the isoforms of adiponectin exhibit different biological functions. Adiponectin concentration is inversely associated with insulin resistance, type 2 diabetes mellitus, and dyslipidemia [5]. Anti-inflammatory and antiatherogenic effects are characteristic of adiponectin, although some studies have demonstrated the association of adiponectin with the risk factors for coronary heart disease in obese or overweight patients [72]. It has been shown that the anti-inflammatory effect of this hormone depends on its level in the blood. At the same time, the level of adiponectin is inversely proportional to the level of inflammatory biomarkers, including C-reactive protein and pro-inflammatory cytokines (interleukin (IL)-6 and IL-10, tumor necrosis factor-α (TNF-α)) [73,74].

High levels of adiponectin correlate with increased insulin sensitivity and glucose tolerance [5]. It is known that VPA-induced weight gain is associated not only with increased levels of insulin and leptin, but also with a decrease in adiponectin levels. However, the underlying mechanisms of changes in the adiponectin levels in VPA-MetS remain unknown [73].

#### 3.3.3. Chemerin

Chemerin belongs to the group of adipokines; it is produced by both adipose tissue and the liver. In addition, it is a chemoattractant for immune cells, such as macrophages, and promotes adipocyte differentiation [75]. Chemerin is secreted as an inactive proprotein and turns into a biologically active form after C-terminal proteolytic cleavage. Chemerin and its receptors are mainly expressed in adipose tissue, but chemerin has been shown to play a different role in the pathogenesis of inflammatory and metabolic diseases in many organs, such as the adipose tissue, lungs, skin, cardiovascular system, reproductive tract, digestive tract, skeleton, and joints. Chemerin levels are higher in obesity and diabetes mellitus. Chemerin release correlates with body mass index (BMI), waist-to-hip ratio, and adipocyte volume, and is also associated with insulin resistance. So, it is considered as one of the biomarkers of MetS [5].

The high plasma levels of chemerin in patients may indicate the involvement of this adipokine in the pathogenesis of MetS and its role as an early VPA-MetS biomarker. Overall, clinical data indicate that circulating chemerin levels are elevated in patients with obesity, diabetes mellitus, and cardiovascular disease [57,76].

#### 3.3.4. Ghrelin

Ghrelin is a peptide involved in the regulation of appetite and energy balance [1]. Ghrelin has both peripheral and central effects. It is mainly secreted by X/A-like cells in the stomach [77] and also (to a lesser extent) in the small intestine, kidneys, testis, pancreas, and brain [77]. A wide range of the effects on various tissues and organs of the human body is characteristic of ghrelin. It is involved in stimulating the secretion of lactotrophs, corticotrophs, and insulin. Ghrelin also regulates the functions of the gastrointestinal tract and pancreas, glucose, and lipid metabolism, as well as the functions of the cardiovascular system [5]. It affects behavior and sleep in humans. In the brain, ghrelin and its receptor are best known for their critical role in food intake mediated by neuropeptide Y [78] and agouti-related peptide [77]. Ghrelin characteristically increases food intake and body weight through its orexigenic, adipogenic, and somatotropic properties [79]. In addition, ghrelin plays a regulatory role in growth hormone release [80,81], is involved in learning and memory [82], modulates motivation and reward, and regulates the response to stress. Soon after its discovery in 1999, the interest in ghrelin in the context of epilepsy increased significantly [77]. Ghrelin levels have been shown to be altered in patients with epilepsy, and ghrelin administration in preclinical seizure and epilepsy models has been considered anticonvulsant [77]. However, the exact mechanism of its effects is still to be understood. It has also been proven that the level of ghrelin in the blood (plasma) decreases with obesity and increases with anorexia nervosa in humans [83,84]. A progressive decline in basal ghrelin levels is associated with an increase in BMI [85]. The total plasma ghrelin level in patients with obesity and cardiovascular disease is lower than in non-obese patients [77,86]. This explains the interest in ghrelin as a promising VPA-MetS biomarker.

#### 3.3.5. Leptin

Leptin (LEP) is a 16 kDa cytokine-like peptide and a proteohormone involved in appetite modulation, energy balance, and weight regulation [5,87]. The primary effect of LEP is manifested in the brain, where the receptor is highly expressed in the hypothalamus. It has been shown that leptin suppresses orexigenic pathways, including neuropeptide Y and the peptide associated with agouti, and activates anorexigenic pathways. A decrease in LEP levels leads to weight gain [88]. Through the effect on the hypothalamus, LEP helps to reduce food intake and increase energy consumption [89]. The serum level of LEP in humans during the period of achieving energy balance (maintaining body weight) correlates with the total amount of fat in the body [90]. This hormone also affects the development of peripheral insulin resistance by weakening the effect of insulin on insulin-sensitive cells. On the other hand, LEP can cause insulin resistance by affecting insulin secretion [91]. People with CVDs have higher LEP levels than people without CVDs [92].

Various authors argue that the level of LEP in blood plasma may be a significant biomarker of VPA-MetS and the risk of developing CVDs [5]. So, some studies have shown an increase in the level of BMI, insulin, and neuropeptide Y at the 3rd month of VPA treatment [74]. It is believed that this increase is also related to the level of insulin and LEP in the blood serum or central and peripheral mechanisms induced by VPA [74]. Elevated serum LEP levels play an important role in VPA-associated weight gain in children [74].

#### 3.3.6. Omentin

Omentin is a newly identified adipokine; it is a protein with a body weight of 38 kDa. Omentin is predominantly expressed in visceral adipose tissue and is secreted mainly from stromal vascular cells rather than adipocytes [93]. The expression of omentin decreases in patients with obesity, insulin resistance, and diabetes mellitus. The plasma level of omentin positively correlates with the serum levels of adiponectin and high-density lipoproteins, but its plasma level negatively correlates with BMI, waist circumference, insulin resistance, triglyceride, and leptin levels. Lower levels of omentin in plasma contribute to the development of insulin resistance, type 2 diabetes mellitus, and cardiovascular diseases in obese or overweight patients. In addition, omentin causes the dilation of blood vessels, helping to reduce the risk of arterial hypertension, and weakens angiogenesis induced by C-reactive protein.

So, omentin has been proposed as a MetS biomarker and a biomarker of endothelial dysfunction in patients with MetS [93]. It has been shown that the level of omentin does not change during treatment with valproate [94], but studies on this topic are also singular, requiring the further study of omentin as a potential metabolic biomarker of VPA-MetS. The study of the plasma level of omentin in the future can help to identify high-risk groups of patients with VPA-induced MetS, so that practicing neurologists and psychiatrists can conduct early interventions and in-depth examinations.

#### 3.3.7. Testosterone

Testosterone is a sex hormone and an anabolic steroid. Testosterone is secreted mainly by the testes in males and, to a lesser extent, by the ovaries in female. It is biosynthesized in several stages from cholesterol and converted into inactive metabolites in the liver. Testosterone is involved in lipid homeostasis in tissues such as the liver, adipose tissue, and skeletal muscle [95]. Low plasma testosterone levels are associated with the development of obesity, insulin resistance, and hypercholesterolemia in men. In addition, men with MetS and diabetes mellitus type 2 have a high prevalence of hypogonadism. MetS and low blood (serum and plasma) testosterone levels are independently associated with an increase in all-cause mortality and cardiovascular mortality.

The serum and plasma levels of testosterone are associated with VPA-MetS development. Studies have shown a higher prevalence of MetS in patients with low testosterone levels [95]. Firstly, VPA-induced obesity and hyperinsulinemia can inhibit the synthesis of sex hormone binding globulin and, consequently, the levels of circulating testosterone. Secondly, low total testosterone and sex hormone-binding globulin levels in men correlate with hyperinsulinism and elevated inflammatory cytokines that accompany obesity and an increased waist circumference [96]. However, the results of previous studies are controversial, as there are studies confirming an increase in the testosterone levels in women taking VPA [97,98,99].

#### 3.3.8. Parathyroid Hormone

Parathyroid hormone is a polypeptide that is synthesized and cleaved into its active form in the parathyroid gland [100]. It is involved in the control of blood calcium levels, as well as phosphorus and vitamin D levels. Parathyroid hormone is also associated with the development of CVDs. A positive correlation was shown between the level of parathyroid hormone and MetS among the elderly and in patients with pathological obesity. Although higher levels of this hormone have been associated with an increased risk of cardiovascular diseases, insulin resistance, arterial hypertension, and obesity in a number of studies, there is no consensus on parathyroid hormone as a biomarker of MetS yet.

With the chronic intake of VPA, the risk of bone fractures in patients with epilepsy increases, as a result of a deterioration in bone metabolism [101], which a number of authors associate with VPA-induced disturbances in the formation of parathyroid hormone [102,103]. However, the clinical significance of parathyroid hormone as a biomarker of VPA-induced MetS needs to be clarified.

#### 3.3.9. Thyroid Stimulating Hormone

Thyroid-stimulating hormone (TSH) is produced by the pituitary gland. TSH stimulates the production of thyroid hormones, including thyroxine (T4) and triiodothyronine (T3). Elevated serum levels of TSH are associated with dyslipidemia [5]. In people with serum TSH levels at the upper limit of the normal range (2.5–4.5 mcg/L), the level of biomarkers and anthropometric indicators of obesity were increased, and the level of TG was increased, as well as a high risk of developing MetS. In recent years, there has been increasing evidence that thyroid dysfunction affects the metabolism of lipids and glucose, blood pressure, and BMI, which are associated with various metabolic parameters and can lead to the development or aggravation of the components of VPA-induced MetS.

VPA-induced thyroid dysfunction may interfere with growth and development in children and adversely affect endocrine hemostasis in adults [104]. Elevated serum levels of TSH may also be associated with an increased incidence of VPA-induced obesity [105]. Many publications note that an increased concentration of TSH is associated with the development of MetS [1]. The study by Güngör et al. [106] demonstrated that the levels of thyroid hormones in the blood serum of children with epilepsy aged 15 months to 16 years changed after 12 months of taking VPA compared to the period before the start of therapy. The serum levels of TSH and free triiodothyronine were significantly higher at the 12th month of treatment with VPA and phenobarbital (PB) compared with the start of therapy and with the control group, and subclinical hypothyroidism at the 12th month was detected in 15.2% of children treated with VPA, versus 2.9% of children treated with PB. When compared with the values before treatment, there was a statistically significant difference in the frequency of subclinical hypothyroidism in the VPA group and there was no significant difference in the PB group. The results of this study are interesting because VPA inhibits the microsomal enzyme systems of the liver. Although, other researchers have shown that enzyme-inducing liver antiepileptic drugs such as PB, carbamazepine (CMZ), and phenytoin (PH) induced the P450 liver enzyme system, resulting in decreased serum thyroxine (T4) and free thyroxine (fT4) levels [104]. Nevertheless, it is recognized that the thyroid function in patients taking VPA for a long time should be monitored regularly.

Glucuronosyltransferase (UGT) may also be responsible for glucuronidation and play a role in thyroid hormone metabolism, as high levels of UGT have been observed following VPA exposure in some studies [104]. Because VPA is an established liver inhibitor, it may have some effect on thyroid hormone metabolism. However, the mechanisms of VPA-induced thyroid dysfunction have not been well established. So, a VPA-mediated decrease in serum T4-binding globulin concentration, a shift away from protein binding sites, and a decrease in T4 5′-deiodinase activity are all possible explanations. In addition to the GABA stimulatory properties of VPA, GABA can inhibit the release of somatostatin, which inhibits TSH secretion. VPA can also lead to a deficiency of zinc and selenium, which play an important role in the synthesis of thyroid hormones. A significant effect of VPA on serum T4 and TSH was shown. T4 was significantly reduced while TSH levels were increased. (T4: SMD = −0.384; 95% CI, −0.5989 to −0.199; TSH: SMD = 0.942; 95% CI, 0.664–1.20) [104,107]. In patients taking VPA, the thyroxine and free thyroxine levels were significantly reduced, while the thyroid-stimulating hormone (TSH) levels were significantly elevated during 6, 12, and 24 months of VPA therapy [108]. However, there has been insufficient research on the relationship between VPA mono-therapy, elevated serum TSH levels, and VPA-MetS. Nevertheless, it is recognized that the thyroid function in patients taking VPA for a long time should be monitored regularly. This biomarker of VPA-MetC may be a promising.

### 3.4. Other Organic Compounds

#### Direct and Total Bilirubin

Bilirubin is a bile pigment that is formed during the breakdown of hemoglobin, or rather heme—an iron–containing protein in the composition of hemoglobin contained in red blood cells. At the same time, the metalloprotein hemoglobin is released from the dead cells, consisting of an iron-containing part, heme, and a protein component, globin. Iron is split off from heme, which is reused as a necessary component of enzymes and other protein structures, and heme proteins are converted into bilirubin. Indirect (unconjugated) bilirubin with the help of albumins is delivered by the blood to the liver, where, thanks to the enzyme glucuronyltransferase, it combines with glucuronic acid and forms direct (conjugated) bilirubin. The bound fraction of the pigment practically does not enter the blood and is normally excreted with bile. Bilirubin is a potentially toxic metabolite [109]. However, it has antioxidant and anti-inflammatory properties, including the ability to absorb ROS and inhibit LDL oxidation. In addition, bilirubin is involved in suppressing the expression of cell adhesion molecules, vascular cell adhesion molecules 1 (VCAM-1), and intercellular adhesion 1 (ICAM-1) in vitro [110]. A moderately elevated level of bilirubin is negatively associated with the development of cardiovascular diseases and other diseases mediated by oxidative stress [1]. Low serum direct bilirubin is associated with an initial and subsequent CVD diagnosis, MetS criteria at baseline, and rapid glucose criteria at follow-up. The serum level of indirect bilirubin is associated with the MetS criteria only at the initial stage of the development of this disorder. Bilirubin intake increases with pathological obesity and MetS due to increased oxidative stress. This, in turn, leads to a decrease in the serum level of bilirubin, the development of endothelial dysfunction, and an increased risk of CVDs, since the antioxidant properties of bilirubin weaken. There is a relationship between the serum level of direct bilirubin and the risk of CVDs [111,112,113]. The causes of valproate-induced hepatotoxicity are active [14]. The results of these studies demonstrate that VPA-reactive (toxic) metabolites inhibit the mitochondrial β-oxidation pathway of many endogenous and exogenous compounds in the liver. These metabolites increase the formation of ROS and cause excessive oxidative stress. In addition, there is no doubt about the genetic predisposition of valproates to this ADR, including polymorphisms of the genes of mitochondrial carbamoyl phosphate synthase I (the *CPS1* gene), mitochondrial polymerase gamma (the *POLG* gene), glutathione S-transferase (the *GST* gene), mitochondrial superoxide dismutase 2 (the *SOD2* gene), uridine diphosphate glucosyltransferase (the *UGT* family genes), and genes encoding key cytochrome P450 (CYP) isoenzymes involved in the metabolism of VPA and its reactive metabolites in the liver (for example, the *CYP2C9* gene) [114]. In addition, VPA reduces the binding of bilirubin to plasma proteins and slows down the rate of its excretion from the human body. The combined effect of these mechanisms may explain the prospects for using the levels of direct and indirect bilirubin as a metabolic biomarker of VPA-MetS.

### 3.5. Proteins

#### 3.5.1. Adipocyte Fatty Acid Binding Protein

Adipocyte fatty acid binding protein (A-FABP) is a small lipid-binding protein that is an adipokine. It is produced predominantly by adipocytes, but is also produced by macrophages and endothelial cells [115]. The association between a MetS diagnosis and serum A-FABP levels had a sensitivity of 40% and a specificity of 99% at a protein level of 16 mg/L [1]. Usually, the patients with familial combined hyperlipidemia and non-alcoholic fatty liver disease have high serum levels of A-FABP [115,116]. Increased serum levels of A-FABP are also associated with left ventricular diastolic dysfunction in MetS-comorbid obesity and cardiometabolic disorders [117]. This biomarker may be a prognostic factor for the psychopharmacotherapy-induced MetS development [5], including VPA-MetS [118]. These biomarkers have been known for many years but are still not fully understood. A critical mechanism for VPA-induced steatosis and fatty liver may be the VPA-induced inhibition of enzyme carnitine palmitoyltransferase Iα (CPT1α) [113]. Additionally, CD36-dependent lipid uptake, TG synthesis, and lipid droplet formation are involved in VPA-induced steatosis [118]. A repeated dosing of VPA modulates both the gene expression and DNA methylation levels of the PPARα gene, the PPARγ gene, and the CD36 gene in hepatocytes in humans [119]. Thus, the results of previously conducted studies demonstrate the complex mechanisms of the involvement of VPA in the development of fatty liver disease and an increase in serum levels of A-FABP [118]. So, A-FABP may become a new metabolic biomarker for VPA-MetS in the future.

#### 3.5.2. C-Peptide

C-peptide is a polypeptide formed when proinsulin is cleaved by peptidases. Together with the formed insulin, C-peptide is secreted into the bloodstream [120]. It is produced in equal molar amounts relative to insulin, mainly by the kidneys. C-peptide has a half-life 3–4 times longer than that of insulin [120]. It is used to diagnose insulin-dependent diseases and may be a biomarker for VPA-MetS monitoring [121]. Since there is one molecule of C-peptide for every insulin molecule, it is a good biomarker for assessing the amount of endogenous insulin. The level of C-peptide is higher in patients with hyperglycemia and diabetes mellitus [122]. The role of C-peptide is considered as a prognostic biomarker of cardiovascular pathologies [74,123] in patients with VPA-MetS.

#### 3.5.3. Cystatin-C

Cystatin-C (cys-C) is a 15 kDa protein. All cells with a nucleus produce cys-C, and in humans it is found in almost all tissues and body fluids. This protein acts as a negative regulator of pro-atherogenic cysteine proteases [124] and is considered as a potential sensitive biomarker of changes in glomerular filtration in the kidneys, SVDs, and MetS [5,125,126,127,128], because cys-C is a powerful inhibitor of cysteine protease, which plays a pleiotropic role in the pathophysiology of human vessels. Patients with high serum cis-C levels are at the greatest risk of CVDs (even with mild renal dysfunction), and patients with very high serum cis-C levels usually have arterial hypertension, dyslipidemia, high BMI, and higher serum levels of C-reactive protein. The elevated serum levels of circulating cis-C is associated with the risk of atherosclerosis, coronary heart disease, stroke, and MetS. This association does not depend on kidney function, determined by formulas based on creatinine or other risk factors for the development of SVDs and MetS. However, the role of cys-C as a metabolic biomarker for VPA-MetS development needs further research.

#### 3.5.4. Ferritin

Ferritin is the main iron storage protein. It is necessary for iron homeostasis and participates in a wide range of physiological and pathological processes in the human body. It is synthesized in hepatocytes [129]. The serum ferritin level is mainly used as a biomarker of total iron reserves in the body. High serum ferritin levels are associated with SVD, coronary heart disease, cancer, and adverse outcomes after stem cell transplantation. In addition, studies describe new functions of ferritin independent of iron accumulation, including its relationship with the development of MetS and hereditary metabolic diseases (familial combined hyperlipidemia and familial hypertriglyceridemia) [130]. The risk of myocardial infarction in men with serum ferritin levels > 200 mcg/L was 2.2 times higher than in men with normal serum levels of this biomarker. At the same time, the positive correlation between serum ferritin levels and the risk of myocardial infarction is higher in current or former smokers.

However, the results of previous studies are not unambiguous, since some studies have shown no change in ferritin levels against the background of developed VPA-induced anemia [131,132] and aplastic syndrome [133], while other studies have demonstrated an increase in ferritin levels after 2–3 years of taking valproates, especially in men over 60 years of age [132,134]. So, the levels of ferritin as a metabolic biomarker for the development of VPA-MetS requires further study.

#### 3.5.5. Fibrinogen

Fibrinogen is a soluble plasma glycoprotein synthesized by the liver. It is converted by thrombin to fibrin during blood coagulation [135]. High plasma fibrinogen levels contribute to an increased risk of CVDs in people with diabetes [136]. Hyperfibrinogenemia has long been considered as a MetS biomarker [137].

ADRs such as thrombocytopenia have been reported with VPA; abnormal platelet function; a decrease in the concentration of von Willebrand factor; abnormal bleeding time; and activated partial thromboplastin time (APTT) with reduced fibrinogen (Fbg) and prolonged prothrombin time (PT) [135,138]. The level of fibrinogen is considered as a clinically significant metabolic biomarker of VPA-MetS, which is important to consider in real clinical practice.

#### 3.5.6. Fibroblast Growth Factor 21

Fibroblast growth factor (FGF-21) is a polypeptide produced predominantly in liver tissues [139]. It has a beneficial effect on glucose and lipid metabolism and has a high sensitivity to insulin [1]. The serum levels of FGF-21 were significantly higher in overweight/obese people than in normal or underweight people. FGF-21 levels were positively correlated with obesity, fasting insulin, and triglycerides and negatively correlated with LDL levels [140]. VPA is known to have potential ADRs such as hepatotoxicity. FGF21 is a functional cytokine for metabolic regulation [141], including in patients taking VPA. Serological evidence suggests that patients with epilepsy treated with VPA also have markedly elevated levels of FGF21 in plasma samples [141]. In patients with bipolar affective disorder, FGF21 levels were significantly elevated after VPA treatment, and high FGF21 levels were significantly correlated with treatment outcome and the development of MetS. These results suggested that FGF21 may be a common metabolic biomarker for the development of VPA-MetS [142,143].

#### 3.5.7. Monocyte Chemoattractant Protein-1

Monocyte chemoattractant protein-1 (MCP-1) is one of the key chemokines that regulates the migration and infiltration of monocytes/macrophages. It is secreted by adipocytes. Both MCP-1 and its receptor have been demonstrated to be induced and involved in various diseases. MCP-1 plays an important role in atherosclerosis development [144]. Diabetic patients and patients with MetS have high levels of MCP-1; it is associated with a mild systemic inflammatory response [145,146]. Experiments have shown a significant increase in the level of monocytic chemoattractant protein-1 on VPA therapy [147]. Other studies have shown the inhibition of MCP-1 by valproates [148]. These results demonstrate that MCP-1 can be considered as a potential metabolic biomarker for VPA-MetS.

#### 3.5.8. Plasminogen Activator Inhibitor-1

Plasminogen activator inhibitor-1 (PAI-1) is a serpin-containing glycoprotein that is secreted by several cell types [149]. PAI-1 can bind to free and receptor-bound urine plasminogen activator (uPA), thereby inhibiting uPA-mediated degradation of the extracellular matrix. PAI-1 is an inhibitor of the fibrinolytic system, sometimes referred to as a prothrombotic adipokine [5]. High levels of PAI-1 are associated not only with thrombosis and fibrosis, but also with obesity and insulin resistance. In addition, high plasma levels of PAI-1 is a common feature in patients with MetS, CVDs, and is directly related to the severity of the diseases [150]. Treatment with VPA reduces the plasma levels of PAI-1 and alters the fibrinolytic balance, measured as t-PA activator inhibitor/Plasminogen-1 ratio (t-PA/PAI-1), in a profibrinolytic direction. The effect is explained by the fact that VPA is a selective class I histone deacetylase inhibitor and thus affects the epigenetic regulation of gene expression [14,151]. This may partly explain the reduction in the incidence of myocardial infarction with VPA treatment observed in recent pharmacoepidemiological studies [152,153,154]. However, the results obtained are debatable. Research on PAI-1 as a metabolic biomarker for VPA-MetS continues.

#### 3.5.9. Retinol-Binding Protein 4

Retinol-binding protein 4 (RBP-4) is a transport protein for retinol. This protein is produced predominantly in the liver, but is also produced in increased amounts by adipocytes [155]. The RBP4 may contribute to the development of metabolic dysfunction by disrupting adipocyte differentiation and increasing the secretion of pro-inflammatory cytokines by macrophages. RBP4-mediated TNF-α induction may additionally inhibit adipocyte differentiation. Moreover, elevated plasma levels of RBP-4 have been associated with an unfavorable biomarker profile of oxidative stress and inflammation [5]. The LEP increases the level of RPB4 protein in humans. High levels of RBP-4 may be associated with MetS components [5,156]. Individuals with very high levels of RBP4 in their blood have a significantly increased risk of developing MetS. The RBP-4 level correlates with the waist-to-hip ratio or visceral fat area, and can be used as a predictive MetS biomarker [5]. Thus, VPA has been shown to reduce the level of RBP-4 [157], which may indicate a potential role of this metabolic biomarker in the development of VPA-MetS.

#### 3.5.10. Tumor Necrosis Factor Alpha

Tumor necrosis factor alpha (TNF-α) is a pro-inflammatory cytokine that has a pleiotropic effect on various types of cells in the human body. TNF-α is secreted from infiltrated macrophages into adipose tissue and lipocytes. TNF-α has been identified as the main regulator of inflammatory reactions and a participant in the mechanisms of pathogenesis of some inflammatory and autoimmune diseases, SVD, diabetes mellitus, insulin resistance, and MetS [158,159,160]. High serum TNF-α levels are associated with MetS regardless of the components of MetS [5]. However, the results of previous studies on VPA-MetS are mixed, as some studies have shown that VPA suppressed the production of TNF-α by inhibiting the activation of nuclear transcription factor kappa B (NF-kappaB) [161,162], while in others VPA promoted the release of TNF-α from activated astrocytes, inducing neuronal apoptosis [163]. Thus, TNF-α can be considered as a promising clinically relevant VPA-MetS biomarker in the future.

#### 3.5.11. Neuropeptide Y

Neuropeptide Y (NPY) is expressed in the brain (in a subset of interneurons of the hippocampus and cerebral cortex, as well as in almost all neurons of the reticular thalamus) and autonomic nervous system. Its blood level is associated with mental disorders (anxiety, depression, epilepsy, schizophrenia, cognitive disorders, etc.), sleep disturbance, and metabolic disorders (obesity) [164]. The regulation of NPY is an important modulator of epilepsy and epileptogenesis. The VPA treatment may alter NPY expression in hippocampal cultures [165].

Experiments have shown an increase in mRNA and NPY levels in humans after chronic treatment with VPA at therapeutic concentrations. In addition, NPY upregulation may influence VPA anticancer treatment of neuroblastomas [1], and counteracting hypothalamic NPY effects may help improve VPA-induced weight gain and obesity without interfering with the desired central effects of VPA [164]. In children aged 2 to 10 years, taking VPA for less than two years resulted in a significant increase in NPY levels, which was associated with an increased appetite (primarily affecting carbohydrate intake) and weight gain [166]. This is because as NPY production increases, energy intake increases, and then fat accumulation and body weight increase.

Since NPY plays a role in both seizure control and weight control, NPY is a possible biomarker for the association between VPA use and weight gain as one of the components of VPA-induced MetS. However, it is not yet clear enough whether VPA directly or indirectly affects blood NPY levels, so more research is needed to investigate this relationship. Thus, NPY can be considered as a potential biomarker of VPA-MetS, but there is still no clear answer to the question of its pathogenetic role in the development of VPA-MetS.

### 3.6. Lipids

#### 3.6.1. Apolipoprotein A1

Apolipoprotein A1 (apo-A1) is the main structural protein found in HDL, which is a part of HDL-C and helps to remove cholesterol from the walls of blood vessels. High levels of Apo-A1 are present in plasma and extravascular tissues [167]. It is synthesized in the liver (predominantly) and intestine. The production of ApoA-I regulating factors, including the modulation of the concentration of ApoA-I mRNA in tissues, are important factors determining the concentration of HDL-C in plasma and may contribute to relative resistance to hypercholesterolemia, atherosclerosis, and MetS. Measurements of apo-AI levels have proven to be very useful in diagnosing and monitoring genetic diseases such as Tangier’s disease associated with low HDL-C levels [168]. The ratio of apo-B to apo-A1 is higher in MetS and is a predictive biomarker of MetS and pre-MetS [169], so this biomarker is also promising for diagnosing VPA-MetS in the future.

#### 3.6.2. Apolipoprotein B

Apolipoprotein B (apo-B) is a blood plasma protein that is part of LDL. In addition, apo-B is the main apolipoprotein of chylomicrons, very low density lipoproteins, and its residues. Apo-B is a carrier of “bad cholesterol”, which causes the accumulation of cholesterol in the walls of blood vessels. The first form of Apo-B is synthesized by the liver and promotes the transport of cholesterol from LDL to various tissues. The second form of Apo-B is produced only by the intestine and is involved in the catabolism of chylomicron residues in the liver [170]. There is a significant relationship between the plasma level of apo-B and the development of atherosclerosis [171].

Apo-B concentration may be more predictive of CVDs risk in patients taking VPA than using the traditional metabolic biomarkers VPA-MetS. Furthermore, an association was found between MetS constituents and higher levels of apo-B [172], which explains the increased interest of researchers in this metabolic biomarker.

#### 3.6.3. Free Fatty Acids

Free fatty acids (FFAs) are types of lipids released from adipose tissue and several types of cells during lipolysis. FFAs are non-esterified fatty acids and are released as a result of the hydrolysis of triglycerides (a triglyceride molecule consists of three molecules of fatty acids bound to glycerin) in adipose tissue by lipoprotein lipase. The release of FFAs from adipose tissue decreases when blood flow through the tissue is restricted. FFAs play an important role in energy supply and as structural components, they are participants in various biological processes in the human body. The lipolysis is inhibited by insulin and stimulated by catecholamines, glucagon, and adrenal corticoids [5].

FFAs circulate in the blood in a protein-bound form, serving as an energy source for various tissues of the human body [173]. Chronically elevated FFAs levels are observed in obese people, as well as in patients with diabetes mellitus, insulin resistance, and CVDs. A high level of this biomarker is associated with the risk of sudden death syndrome in patients with coronary heart disease [174,175]. The erythrocyte FFAs profile not only shows an association with MetS, but also correlates with most of its individual components. However, the study of separated FFAs is a more accurate approach to determining their role in MetS risk in patients who take psychotropic drugs for a long time [5]. Being a simple fatty acid, VPA is a substrate for the β-oxidation pathway of FFAs, which occurs primarily in the mitochondria. The toxicity of VPA has long been considered primarily due to its interference with mitochondrial β-oxidation [14,176]. Research results show that VPA can influence the increase in the level of FFAs and contribute not only to the development of fatty liver disease [177], but also to the development of VPA-MetS [178]. At the same time, there are conflicting results from studies where VPA reduced FFAs [179]. Based on this, FFAs can be considered as a clinically relevant metabolic biomarker for VPA-MetS.

#### 3.6.4. Oxidized Low Density Lipoprotein

Oxidized LDL (Ox-LDL) has been attributed to numerous pro- and anti-atherogenic properties. However, Ox-LDL has not been defined or characterized since its components and composition vary depending on the source, and method of obtaining, storing and using biosamples [180]. It contains unoxidized and oxidized derivatives of fatty acids, both in ester and free form, their degradation products, cholesterol and its oxidized products, proteins with oxidized amino acids and cross-links, polypeptides with various degrees of covalent modification by lipid oxidation products, and much more [181]. It is believed that Ox-LDL is associated with cardiovascular risk factors in diabetes mellitus [1]. Elevated levels of circulating Ox-LDL have been found in various cohorts of patients with MetS [1]. Long-term VPA use is associated with metabolic disorders such as weight gain and may be an alteration in lipid profiles that contribute to cardiovascular events; however, these effects have not been fully determined [181]. There is not enough research on this topic; therefore, it requires further study of the role of Ox-LDL as a metabolic biomarker of VPA-MetS in the future.

#### 3.6.5. Cholesterol

Cholesterol is an organic compound, a natural fatty (lipophilic) alcohol contained in the cell membranes of all living organisms [182]. The term cholesterol includes total cholesterol (TC), TG, and HDL-C (or the TC/HDL-C ratio). The higher the TC/HDL-C ratio, the higher the risk of CVDs. In most cases, it is desirable that the ratio of patients should be below 5:1. The ratio below 3.5:1 is considered very good. Dyslipidemia is a violation of the normal (physiological) ratio of blood lipids. With prolonged existence, dyslipidemia leads to the development of atherosclerosis and SVDs, increasing the risk of developing such serious outcomes (myocardial infarction and ischemic stroke). Dyslipidemia includes an increase in chylomicrons, very LDL (VDL), LDL, apo-B and TG-containing lipoproteins, and low levels of HDL-C [5]. The use of VPA is associated with metabolic rearrangements, including changes in lipoproteins. At the same time, long-term VPA treatment reduces the level of total cholesterol in adults. However, the effects of valproates on total cholesterol are still being discussed. The role of VPA-MetS in LDL as a significant biomarker needs to be clarified.

#### 3.6.6. High Density Lipoprotein

High-density lipoprotein (HDL-C, known as ApoA-I-containing lipoprotein) is a key mediator in reverse cholesterol transport, which is the process of moving cholesterol from extrahepatic tissues back to the liver. HDL heterogeneity is the result of the activity of several factors that collect and reconstruct HDL particles in plasma. Low HDL-C cholesterol is closely associated with an increased risk of CVDs and MetS; the risk increases by 2–3% with each decrease in the level of HDL-C per mg/dL [183].

Some studies have shown that during the first month of VPA treatment, the mean BMI in patients increased significantly and the mean HDL-C levels decreased significantly [184]. Overall, HDL-C can be considered as a clinically relevant metabolic biomarker for VPA-MetS.

### 3.7. Enzymes

#### 3.7.1. Superoxide Dismutase

Cu/Zn superoxide dismutase (SOD1) is an advanced antioxidant enzyme of the metalloproteinase group; this enzyme converts a superoxide radical into molecular oxygen and hydrogen peroxide through redox reactions. It forms the front line of defense against damage caused by ROS. In addition, SOD1 affects the activation of nuclear gene transcription or as an RNA-binding protein [5]. The SOD1 is an important component of antioxidant defense mechanisms [185].

The SOD1 is negatively correlated with MetS components. Impaired microRNAs can directly or indirectly affect the expression and/or activity of SOD1 associated with inflammation, insulin sensitivity, and lipid metabolism, thus contributing to the progression of MetS. There are various data on this, most of which show that reduced SOD1 levels are determined in people with MetS [186]. Other studies show that VPA inhibits SOD1 activity and thus serves as a mechanism by which VPA exposure increases ROS availability and alters intracellular redox states and may promote the development of VPA-MetS [187].

#### 3.7.2. Gamma-Glutamyl Transferase

Gamma-glutamyl transferase (GGT) is a cell surface protein that promotes extracellular catabolism of glutathione. This protein is located on the outer surface of the cell membrane of many tissues, mainly in the liver, kidneys, and pancreas [188]. In serum, GGT is transported primarily by lipoproteins or albumin. The serum level of GGT depends on several factors: alcohol consumption, body fat content, lipoprotein and glucose levels in blood plasma, and the use of various drugs [1]. Elevated serum GGT is a biomarker for the development of cardiovascular disease. Elevated GGT levels are closely associated with hepatic steatosis [189], and steatosis is associated with cardiovascular disease [190]. An association between elevated serum GGT levels and arterial hypertension [190], and an increased risk of type 2 diabetes mellitus [191] has also been confirmed. An increase in serum GGT largely reflects the presence of ectopic liver fat or secondary inflammation in general. GGT is used as a sensitive biomarker of increased oxidative stress [186]. VPA has been shown to increase serum GGT levels [188]. Therefore, GGT can be considered as a clinically significant metabolic biomarker for VPA-MetS.

#### 3.7.3. Lipoprotein-Associated Phospholipase A

Lipoprotein-associated phospholipase A (Lp-PLA 2) is an enzyme that belongs to the group of intracellular and secretory phospholipase enzymes that can hydrolyze the sn-2 phospholipid ether bond of cell membranes and lipoproteins. The Lp-PLA2 is formed by macrophages and foam cells and is mainly associated with LDL particles in the blood [192]. This enzyme inactivates the known pro-inflammatory mediator PAF-AH. Second, Lp-PLA2 hydrolyzes oxidatively modified polyunsaturated fatty acids, producing lysophosphatidylcholine (LysoPC) and oxidized non-esterized fatty acids (OxNEFA) [192]. Serum levels of ferritin, LDL cholesterol, and apo-B100 affect the enzymatic activity of Lp-PLA 2 [5]. This biomarker is particularly useful for assessing the risk of MetS, diabetes mellitus, and SVDs. The plasma level of Lp-PLA2 belongs to highly specific biomarkers of vascular inflammation, it has low biological variability, and plays an important causal role in the inflammation of atherosclerotic plaque. The inhibition of Lp-PLA2 levels is associated with a decrease in serum levels of pro-inflammatory cytokines.

So, this biomarker is proposed for use in clinical practice to improve the assessment of cardiocerebrovascular risk, especially in patients with MetS (obese patients with mixed dyslipidemia, hyperglycemia, insulin resistance, and arterial hypertension), but research on the interaction between valproate and Lp-PLA 2 is not enough to suggest it as a VPA-MetS biomarker.

#### 3.7.4. Amylase

Amylase is an enzyme secreted by the pancreas and salivary glands. Amylase in small concentrations is also found in other tissues of the human body [193]. The hydrolysis of glycoside bonds in starch molecules and the conversion of complex carbohydrates into simple sugars are the main targets of amylase action. An elevated serum amylase level may be caused by an increase in its production by the pancreas or extra-pancreatic tissues or a decrease in the rate of clearance [194]. Salivary amylase may be preferentially reduced in obese individuals. Some studies have shown a positive correlation between total amylase and serum VPA. Patients produced a simultaneous increase in total amylase and lipase levels, although none of them had symptoms suggestive of acute or chronic pancreatitis and their ultrasound was normal [195]. Other studies have shown that serum pancreatic amylase activity decreased significantly at 6 and 12 months of VPA treatment, while total serum amylase and lipase activity did not show any significant change at 6 or 12 months of treatment. Non-pancreatic isozyme activity of amylase was significantly higher after 6 and 12 months of treatment. In 13% of cases, the total serum amylase level was slightly elevated after 6 and 12 months of treatment. There was no significant correlation of pancreatic amylase serum levels or non-pancreatic amylase isoenzymes with the serum VPA levels at 6 and 12 months of treatment. Non-pancreatic amylase activity, probably derived from the salivary glands, may be increased in children receiving VPA monotherapy. All subjects remained without clinical symptoms of pancreatitis during the entire study period [195]. The results of these studies demonstrate that serum and salivary amylase can be considered as a biomarker for VPA-MetS.

### 3.8. Vitamins

#### 3.8.1. 25-Hydroxyvitamin D

25-Hydroxyvitamin D is a form of vitamin D produced in the liver by the hydroxylation of vitamin D3 (cholecalciferol) by the vitamin D enzyme 25-hydroxylase and produced in the liver by the hydroxylation of vitamin D_3_ by the vitamin D enzyme 25-hydroxylase [196]. A decrease in 25-hydroxyvitamin D (25(OH)D) and an increase in parathyroid hormone have been associated with both MetS and each of its individual components. It is assumed that the decrease in the level of 25(OH)D in MetS is associated with the binding of 25(OH)D in fat [5], and the increase in parathyroid hormone is considered a compensatory mechanism for low levels of 25(OH)D. Some authors argue that serum 25(OH)D, but not parathyroid hormone, was significantly associated with MetS and its components [5,103]. Insufficient vitamin D levels are associated with an increased risk of cardiometabolic diseases, although the results are contradictory. Among men, the plasma level is 25(OH)D and correlates with serum insulin levels, insulin resistance, and serum TG levels. Women have a plasma level of 25(OH)D and it is inversely correlated with serum insulin, insulin resistance, total cholesterol, LDL-C, and HDL-C. The possibility that the effect of vitamin D on MetS differs depending on gender requires further study. In addition, vitamin D levels fluctuate from month to month.

Studies have shown that VPA monotherapy has a negative effect on vitamin D levels, and VPA-induced vitamin D deficiency does not depend on the region of residence of patients [103,197,198,199]. The level of vitamin D_3_ decreases with an increase in BMI in patients taking long-term VPA. However, no significant differences in the serum level of 25(OH)D_3_ were found depending on the gender of patients with epilepsy and people without epilepsy, as well as depending on the mono- and polytherapy of VPA with other anticonvulsants, as well as on the degree of resistance of epileptic seizures to VPA. On the other hand, most authors recognize that vitamin D levels can be considered as an important biomarker of VPA-MetS.

#### 3.8.2. Vitamin E

Alpha-tocopherol, the main form of vitamin E, acts as an antioxidant vitamin in the human body. A number of studies have reported that the serum concentration of vitamin E in patients with cardiovascular disease is lower than in the control group. This indicates an unbalanced redox status of blood serum and a decrease in the antioxidant capacity of lipids in cardiovascular diseases [5]. Chronic VPA treatment reduces the levels of antioxidants such as vitamin E and glutathione peroxidase, further suggesting a role for oxidative stress in the development of ADRs [200,201]. Based on this, the serum level of vitamin E can be considered as a clinically significant metabolic biomarker of VPA-MetS.

### 3.9. Other

#### CD40 Ligand

The CD40 ligand (CD40L) is a pro-inflammatory mediator that is expressed on CD4+ T cells and is activated by platelets. CD40L is expressed on the vascular endothelium and is elevated in cardiovascular diseases [202]. Plasma CD40L levels are higher in patients with cardiovascular disease and ischemic heart disease [203]. The CD40L–CD40 interaction plays an important role in the cascade of inflammatory and pro-atherothrombotic reactions [5]. VPA reduces the level of sCD40L in plasma samples from HIV-1 infected patients and in washed human platelets, which are the main source of circulating sCD40L. VPA also inhibited the human immunodeficiency virus trans-activator 1 transcription-induced release of sCD40L and platelet factor 4 in C57BL/6 mice [204]. The role of CD40 in the development of VPA-MetS continues to be studied.

## 4. Urinary Biomarkers of Valproate-Induced Metabolic Syndrome

The progression of VPA-MetS produces a continuous and monotonous change in the urinary metabolome, characterized by increasing or decreasing levels of the relevant metabolic biomarkers [205].

### 4.1. Carbohydrates

#### 4.1.1. Glucose

Glucose is the most studied urinary MetS and VPA-MetS biomarker. Normally, urine contains practically no glucose. Its level is 0–0.8 mmol/L. On the other hand, in patients with diabetes mellitus, hyperglycemia may increase glucose filtration, which is a consequence of an overload of the tubular transport capacity of the kidneys [206,207]. Impaired glucose metabolism is a mandatory risk factor for the development of CVDs and MetS, according to the WHO definition [5]. Elevated urinary glucose may be a biomarker of VPA-induced insulin resistance [205,207].

#### 4.1.2. Maltitol

Maltitol is a polyol used as an alternative to sugar recommended for individuals at risk of type 2 diabetes [204]. However, the role of this potential urinary metabolic biomarker VPA-MetS is unclear.

### 4.2. Amino Acids

#### 4.2.1. Aromatic Amino Acids

Aromatic amino acids (AAAs) include phenylalanine, tryptophan, and tyrosine. So, AAAs were associated with the risk of type 2 diabetes in a study involving patients with MetS [5]. In a recent study, AAAs have been proposed as MetS metabolic biomarkers in urinalysis [205], but they have not been investigated in VPA-MetS. However, the role of AAAs in this disorder is interesting and needs to be studied in the future.

#### 4.2.2. Histidine

Histidine is an essential amino acid. It is a precursor to certain hormones (e.g., TSH) and metabolites that play an important role in regulating kidney function, neurotransmission, gastric secretion, and immunity. Histidine has unique acid-base properties and is a universal catalytic residue in many enzymes, as well as for those proteins and enzymes that coordinate metal ions. One example of the participation of histidine as a catalytic residue are serine esterases such as trypsin, chymotrypsin, acetylcholinesterase, and various enzymes in the blood coagulation cascade. It is present in various forms, including free L-histidine, Nα-acetylhistidine, in the human body. Histidine has important anti-inflammatory, antioxidant, and antisecretory effects in the human body. Histidine is excreted in the urine. Its low urine level has been suggested as a potential biomarker of MetS. A decrease in histidine excretion is associated with a change in the level of both endogenous imidazoline ligands and α2-adrenergic receptors, and the development of arterial hypertension [5,205]. The role of histidine as a potential urinary biomarker for VPA-MetS needs further investigation in the future.

### 4.3. Organic Acids

#### 4-Hydroxyphenylpyruvic Acid

As is known, 4-hydroxyphenylpyruvic acid (4-HPPA) is an intermediate in tyrosine metabolism. An increase in 4-HPPA in the blood and urine can be considered as an indicator of tyrosinemia [206]. 4-HPPA is also considered as a MetS biomarker [205,207,208,209], but the role of this biomarker is in the early stages of VPA-MetS research.

## 5. Discussion

As you know, VPA is an anticonvulsant and mood-stabilizing drug for long-term treatment, which is widely used in real clinical practice. However, VPA has a number of metabolic and endocrine side effects such as weight gain, hyperinsulinemia, changes in sex hormones, dyslipidemia, hyperleptinemia, etc. In this regard, the existing, but insufficiently studied, problem of VPA-MetS is increasingly recognized as a real one. The importance of this problem in modern neurology and psychiatry is due to the risk of developing serious cardiovascular and endocrine diseases, the development of which can lead to VPA-MetS. Unfortunately, the early diagnosis of VPA-MetS in real clinical practice is difficult because algorithms for diagnosing this ADR have not been developed and there are no widely recognized panels of biomarkers in biological fluids, primarily in the blood and urine.

The metabolome in patients with VPA-MetS determines their individual sensitivity to the effects of VPA and its active metabolites [14], which can be assessed using blood (serum and plasma) and urinary biomarkers (Table 1 and Table 2).

It is known that the metabolome is a set of chemicals with small molecules in the body, but is also the functional state of the body and provides a multifaceted reading of the total activity of endogenous (cellular) and exogenous (environmental) processes [210].

Changes in the metabolome in patients receiving long-term VPA include a set of changes in the level of metabolic biomarkers in biological fluids and various organs and tissues of the body, including peptides, lipids, amino acids, nucleic acids, carbohydrates, biogenic amines, vitamins, and minerals. Such changes in the metabolome can not only significantly affect the disease phenotype, but also the expected therapeutic response to VPA and significantly affect post-translational processes, including secondary transcriptome and proteome changes in patients with neurological diseases and psychiatric disorders.

**Figure 3:**
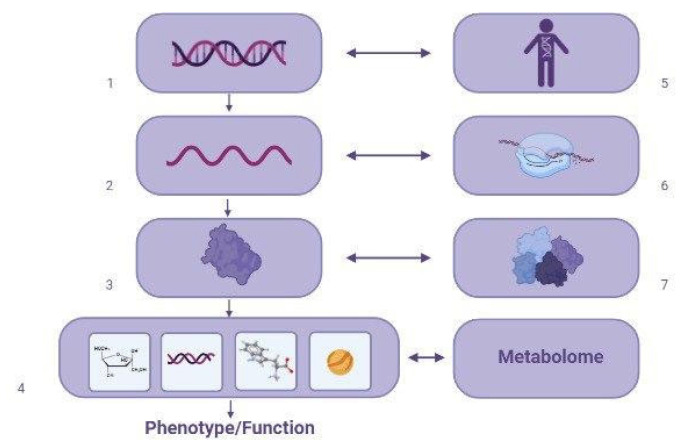
Main factors affecting the metabolome in patients with valproate-induced metabolic syndrome (created with BioRender.com: https://www.biorender.com/ (accessed on 12 April 2023)). Note: 1—DNA; 2—RNA; 3—proteins; 4—metabolites; 5—genome; 6—transcriptome; 7—proteome.

Some components of the metabolome in general, and with VPA in particular, fluctuate rapidly and markedly depending on exposure to biological and environmental factors. Factors known to affect the metabolome include the transcriptome, which includes the set of all transcripts synthesized by a single cell or group of cells, including non-coding ribonucleic acid (RNA); a proteome, which is a set of body proteins produced by a cell, tissue, or organism; and genome, namely a set of hereditary factors [5] (Figure 3), as well as age, sex, drugs, diet, time of day, menstrual cycle, and stress. Depending on metabolome changes, a patient taking VPA may or may not develop VPA-MetS. This review can help the practicing neurologist, psychiatrist, internist, and/or endocrinologist to predict and diagnose VPA-MetS based on the study of blood and urinary metabolic biomarkers, which is of undoubted clinical interest. Although, it should be recognized that VPA-MetS active screening needs further development and a wider application in real clinical practice.

**Figure 4:**
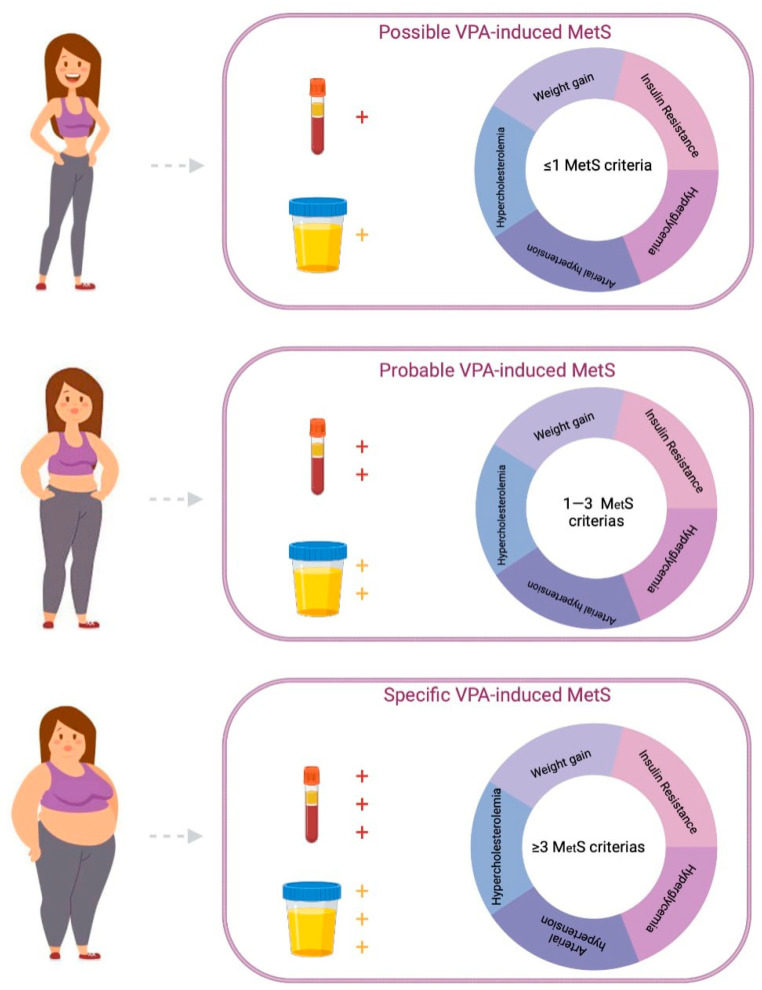
Algorithm for a personalized approach to the diagnosis of valproate-induced metabolic syndrome (created with BioRender.com: https://www.biorender.com/ (accessed on 12 April 2023)).

**Figure 5:**
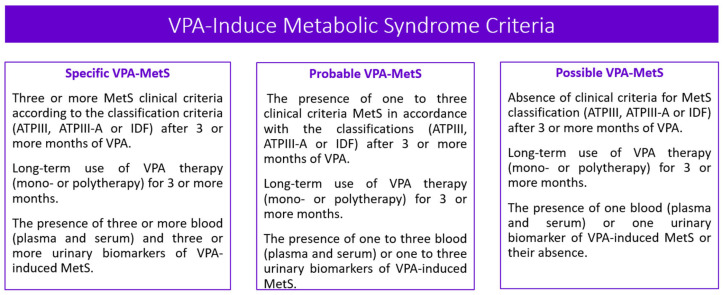
Valproic acid induced metabolic syndrome criteria. Note: VPA—valproic acid; MetS—metabolic syndrome; IDF—the International Diabetes Federation; ATPIII—the Adult Treatment Panel, III edition.

Three types of VPA-MetS development can be distinguished based on the absence or presence of blood (serum and/or plasma) and urinary biomarkers in patients who receive long-term valproate (3 months or more): certain, probable, and possible (Figure 4 and Figure 5).

**Figure 6:**
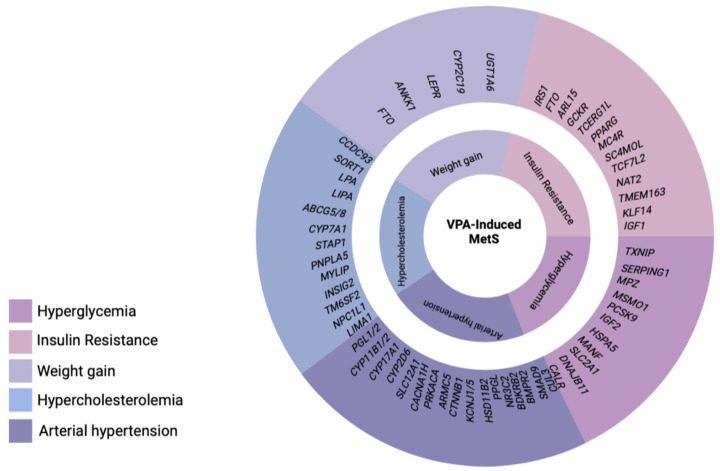
Perspective genetic biomarkers of valproate-induced metabolic syndrome [211,212,213] (created with BioRender.com: https://www.biorender.com/ (accessed on 12 April 2023)).

In addition, a genetic predisposition to both the development of VPA-MetS and the development of some of its components, including weight gain, arterial hypertension, hypercholesterolemia, and hyperglycemia, plays an important role in changing the metabolome in patients taking valproates for a long time. Through the study of the genetic biomarkers of VPA-MetS at the initial stage of its development, although based on the results of previous studies and information in open databases, it is possible to depict the contribution of the most studied candidate genes that are probably associated with a high risk of developing VPA-MetS (Figure 6). Undoubtedly, the study of these genetic biomarkers, along with the metabolic biomarkers of VPA-MetS presented in this review, will become a new direction of “pharmacogenetic informed pharmacometabolomics” [14] in the future.

**Figure 7:**
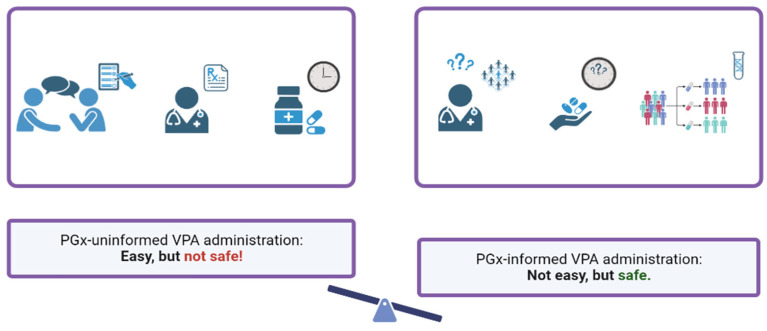
The role of pharmacogenetic testing (PGx) in the study of genetic biomarkers of valproate (VPA)-induced metabolic syndrome (created with BioRender.com: https://www.biorender.com/ (accessed on 10 May 2023)).

The pharmacogenetic testing (PGx) of the non-functional and low-functional alleles of single-nucleotide variants of genes encoding key enzymes of P-oxidation, acetylation (mitochondrial oxidation or beta-oxidation), and glucuronidation of VPA may provide another clue to the genetic predisposition to the development of VPA-MetS due to the accumulation of VPA and its active metabolites in the blood, despite the intake of low and medium doses of this anticonvulsant [14,16] (Figure 7).

In general, future trials are needed to: (i) establish the effect of the long-term use of VPA on the prevention and risk of developing MetS, depending on the age and gender of patients; (ii) compare the long-term effects of VPA on changes in the blood (serum and plasma) biomarkers of MetS, as well as the potential synergistic or antagonizing effect presented in this narrative review of VPA-MetS biomarkers and VPA metabolites (therapeutic and toxic) in the human body on the metabolic effects caused by VPA; (iii) continue to study the mechanisms by which various laboratory diagnostic methods can increase the sensitivity and specificity of VPA-MetS biomarkers and help the practitioner (neurologist, psychiatrist) to prevent the development of VPA-MetS in patients; (iv) to study the potential of environmental factors and lifestyle (nutrition, physical activity, smoking, etc.) of patients taking VPA, which may further increase or decrease the risk of developing VPA-MetS; and (v) to give an idea of genetic and non-genetic factors and tips that will help to optimize a personalized approach to the long-term use VPA and the monitoring of VPA-MetS in patients with neurological diseases and mental disorders, depending on their individual risk.

## 6. Limitation

In general, we have not found large multicenter studies of VPA-MetS or a sufficient number of single-center studies with a similar design. Other limitations of this narrative review are the lack of information about the frequency of occurrence of VPA-MetS. This is due to the difference in the design of the studies we analyzed, including: (a) the age of the patients (children and/or adults), which significantly affects not only the metabolic rate and elimination of VPA, but also the risk of drug-induced metabolic disorders; (b) the gender of patients, which can affect the rate of fatty acid metabolism, including VPA (although a number of studies have shown that female sex increases the risk of developing VPA-MetS); (c) the type of psychopharmacotherapy (monotherapy with valproates, co-administration with other anticonvulsants, co-administration with antipsychotics, or co-administration with normotimics), which can affect the metabolism of VPA due to drug interaction and thereby increase or decrease the level of its active metabolites in the blood; (d) the duration of taking VPA (from 1 year to several years), which undoubtedly can affect the frequency of development of VPA-MetS; (e) the dose of VPA varied depending on the age of patients and neurological disease (more often, epilepsy) or mental disorder (more often, bipolar disorder and schizophrenia), although a high dose of valproates probably has the greatest adverse effect on metabolic processes in the human body and the risk of developing VPA-MetS; (f) therapeutic drug monitoring was not carried out in most of the publications we analyzed, although peak and residual (minimal) levels of VPA in the blood (serum and plasma) may affect changes in the level of the VPA-MetS biomarkers presented in this review; and (g) the genetic predisposition that can affect both the frequency of development of the components and the frequency of VPA-MetS in general. Undoubtedly, all of the above limitations of this review indicate the need for further study of the problem of VPA-MetS in the future and explain the need for large multicenter studies of this ADR with a single and well-planned design. We recognize that there are currently more questions about VPA-MetS than there are answers.

## 7. Conclusions

The development of new technologies for the laboratory diagnostics of recent decades and their introduction into clinical practice allow not only a fresh look at the pathophysiological mechanisms of metabolic disorders in patients with neurological diseases and mental disorders taking valproates for a long time, but also to help significantly expand the monitoring capabilities of specific, probable, and possible VPA-MetS. One of the important aspects mentioned in recent studies, but not discussed in this narrative review, is a genetic predisposition that affects VPA and the symptoms of VPA-MetS (obesity, arterial hypertension, hyperglycemia, insulin resistance, and hypercholesterolemia). Obviously, not all patients with neurological diseases and mental disorders taking valproates will have VPA-MetS. However, the study of the patient’s metabotype, based on the analysis of blood (serum and plasma) and urinary biomarkers of VPA-MetS, will optimize therapeutic strategies for the management of patients with neurological diseases and mental disorders in the future.

## Figures and Tables

**Table 1:** Potential Serum and Plasma Biomarkers of Valproate-Induced Metabolic Syndrome.

Biomarker	Reference Values	Change in MetS	Symptom of MetS	References
A. Carbohydrates				
Glucose	7.19 ± 0.94 µmol/L	Controversial	Insulin resistance	[53,54,55,56,57]
B. Acids				
Uric acid	335.67 ± 94.77 µmol/L	Controversial	CVD	[57,58,59,60,61,62,63]
C. Hormones				
Adiponectin	13,086.6–14,196.7 ng/mL	Low	Insulin resistance	[57,70,71,72,73,74]
Chemerin	78.12–112.10 ng/mL	High	CHD	[57,75,76]
Ghrelin	110.2 ± 41.23 ng/mL	High	Obesity	[57,77,78,79,80,81,82,83,84,85,86]
Insulin	0.85–1.7 µmol/L	High	Insulin resistance	[57,64,65,66,67,68,69]
Leptin	8.54–14.4 ng/mL	High	Insulin resistance Leptin resistance	[74,87,88,89,90,91,92]
Omentin	140.19 ± 7.35 ng/mL	Normal	CVD	[57,93,94]
Parathyroid hormone	15–65 ng/mL	High	CVD	[57,100,101,102,103]
Testosterone	M 270–1070 ng/dL F 15–70 ng/dL	Controversial	Obesity	[57,95,96,97,98,99]
Thyroid stimulating hormone	0.4–5.5 µIU/mL	Controversial	CVD	[57,104,105,106,107,108]
D. Other organic compounds				
Bilirubin direct and total	2.5–9 µmol/L	High	CVD	[57,109,110,111,112,113,114]
E. Proteins				
Adipocyte fatty acid-binding protein	20.2–32.3 microg/L	High	Obesity cardiometabolic disorders	[57,115,116,117,118]
C-peptide	1.1–0.9 ng/mL	Controversial	Insulin-related diseases	[57,120,121,122,123]
Cystatin C	0.5–0.89 ± 0.23 mg/L	High	Cardiometabolic disorders	[57,124,125,126,127,128]
Ferritin	M 20–250 µg/L, F 10–120 µg/L	Controversial	Oxidative stress Cardiometabolic disorders	[57,129,130]
Fibrinogen	0.53–1.6 g/L	Low	Bleeding	[57,135,136,137,138]
Fibroblast Growth Factor 21	107.44 ± 18.07–204.19 ± 53.57 ng/mL	High	Obesity CHD	[57,139,140,141,142]
Monocyte chemoattractant protein-1	300– 500 pg/mL	Controversial	CHD	[57,144,145,146,147,148]
Plasminogen activator inhibitor-1	1–24 E/mL	Low	Profibrinolytic effect	[57,149,150,151,152,153,154]
Retinol-binding protein 4	6–400.0 ng/mL	Low	CVD	[57,155,156,157]
Tumor necrosis factor-a	<8.1 ng/mL	Controversial	CHD	[57,158,159,160,161,162,163]
Neuropeptide Y	16 nM	High	Hyperinsulinemia	[57,164,165]
F. Lipids				
Oxidized low density lipoprotein	26–117 IU/L	Controversial	CVD	[57,180,181]
Cholesterol	3–6 mmol/L	Controversial	CVD	[57,182]
Apolipoprotein A1	0.99 ± 0.29 g/L	Low	CVD	[57,167,168,169]
Apolipoprotein B	0.97–0.09 g/L	Low	CVD	[57,170,171,172]
Free fatty acids	M 8.3–10.9 ng/mL, F 11.4–13.6 ng/mL	High	Insulin resistance	[57,173,174,175,176,177,178,179]
High density lipoprotein	2.89–0.23 mmol/L	Low	Insulin resistance	[57,183,184]
Low-density lipoprotein cholesterol	0.94 ± 0.23 mmol/L	Low	CVD	[57]
Triglycerides	3.19–0.21 mmol/L	High	Dyslipidemia Obesity	[57]
G. Enzymes				
Superoxide Dismutase	1200–2000 U/g	Low	Oxidative stress Inflammation	[57,185,186,187]
Gamma-glutamyl transferase	M 10–71 U/L, F 6–42 U/L	High	Oxidative stress Inflammation	[57,188,189,190]
Lipoprotein-associated phospholipase A	<200 ng/mL	Controversial	CVD	[57,192]
Amylase	28–85 U/L	Controversial	Oxidative stress Inflammation	[57,193,194,195]
H. Vitamins				
25-Hydroxyvitamin D	30–100 ng/mL	Low	CVD Inflammation	[57,194,195,196,197,198,199]
Vitamin E	5.00–18.00 µg/mL	Low	Oxidative stress Inflammation	[57,200,201]
I. Other				
Ligand CD40	N/A	High	CVD Diabetes mellitus	[202,203,204]

Note: M—male, F—female, AH—arterial hypertension, BMI—body mass index; CVD—cardiovascular disease, CHD—coronary heart disease.

**Table 2:** Potential Urinary Biomarkers of Valproate-Induced Metabolic Syndrome.

Biomarker	Reference Values	Change in MetS	Symptom of MetS	References
A. Carbohydrates				
Glucose	0–0.8 mmol/L	High	Insulin resistance	[57,205,206,207]
Maltitol	None	High	Insulin resistance	[57,205]
B. Amino acids				
Aromatic amino acids	None	High	DM 2	[57,205]
Histidine	52–162 µmol/mmol	Low	AH	[57,205]
Tryptophan	0.4–1.4 mg	High	CVD	[57]
C. Acids				
4-hydroxyphenylpyruvic acid (4-HPPA)	None	High	Insulin resistance	[57,205,206,207,208,209]

Note: None—typically should not be detected in the urine; MetS—metabolic syndrome; AH—arterial hypertension; BMI—body mass index; CHD—coronary heart disease; CVD—cardiovascular disease; DM 2—diabetes mellitus 2.

## Data Availability

Not applicable.
